# Kathryn Chu: delivering surgery as part of primary health care

**DOI:** 10.2471/BLT.20.030120

**Published:** 2020-01-01

## Abstract

Kathryn Chu talks to Gary Humphreys about the importance of surgical care in primary health care delivery.

GH: How did you come to study medicine?

KC: I did my bachelors in human biology at Stanford University but hadn’t planned on going to medical school. During my junior year abroad, I interned at a women’s hospital in Switzerland which inspired my interest in medicine, but it was only later in Honduras that surgery really got under my skin. I had already been at the University of California-San Francisco, School of Medicine for three years at that point, but I still hadn’t decided on a career path.

GH: What happened in Honduras?

KC: I was working in a small clinic on the Mosquito Coast which was cut off from other health facilities by a lagoon. As I said, I was a medical student at the time and didn’t have any training as a surgeon. I was assisting a woman with a fairly straight-forward vaginal delivery. It all seemed to be going well but then she started bleeding and we couldn’t stop it. She bled to death in less than an hour. There were no doctors around, just myself and the nurses. We tried everything we could, including uterine massage. She was a young woman, probably around 30 years old. Watching her die, not knowing what to do, really made an impression on me. Fortunately, I had more positive experiences that year also, working at mission hospitals in Kenya and Lesotho. They also had limited resources, but incredible surgical providers doing great work in difficult conditions. So, I came back from that year with a different attitude towards surgery – a sense of its fundamental, life-saving importance, but also a different view of how it fits into the broader spectrum of health care delivery.

GH: What do you mean by that?

KC: Prior to those experiences, I’d seen surgery as a high-tech, specialized field that had to be delivered in tertiary-level hospitals. In Latin America and subsequently in Africa I saw that life-saving surgical procedures could be performed at the primary health-care level, without fancy equipment or specialist doctors. Some of the remote hospitals I worked in didn’t have surgeons, only family doctors, general practitioners, and clinical officers who had been working there for decades. And they performed essential and emergency surgical procedures with good outcomes.

“Life-saving surgical procedures [can] be performed at the primary health-care level, without fancy equipment or specialist doctors.”

GH: You took time out from your career as a surgeon in 2002 to go to the London School of Hygiene and Tropical Medicine where you did a masters degree in public health in developing countries. What inspired that decision?

KC: I was conscious that I lacked a broader public health perspective. At the time, there weren’t many mechanisms for surgeons to study public health, but then I was lucky enough to get a scholarship that allowed me to do the masters in London. I met a lot of great people there, including from *Médecins Sans Frontières* (MSF) who were delivering health care in some challenging conflict and disaster settings. It was a fascinating year for me, a year of making connections between surgery and public health and of getting to grips with the challenges around health system strengthening. Remember, this was before the academic field of global surgery was defined.

GH: How would you define the field?

KC: I would say it comprises the activities and research directed towards improving health and health equity for people needing surgical care. The field focuses on underserved populations in countries of all income levels, as well as populations in crisis, like those experiencing conflict or natural and man-made disasters.

GH: You later worked with MSF and are currently vice-president of MSF-Southern Africa. How did that association come about?

KC: In 2007, MSF opened a medical unit based in South Africa and I applied for the job. I was in the United States of America (USA) at the time and really eager to get back to sub-Saharan Africa. MSF were looking for surgeons, but they were also looking for an epidemiologist for operational research. So, I was offered the job and took it. Not everybody understood the decision at home. I made a fraction of the salary I’d been making in the USA, but I knew it was the right decision and the job turned out to be life-changing for me.

GH: In what way?

KC: Different ways. For example, working in academia I was often one of a number of surgeons, whereas in Africa I was often the only surgeon while I was on missions and I felt that I was really making a difference. I was also doing some interesting research helping MSF define minimum standards for safe humanitarian surgery.

GH: Can you talk about that a little?

KC: There was some concern at the time about aid organizations performing surgery during disasters and emergencies without proper drugs and equipment and without due regard for best practice, such as amputating legs without anaesthesia during the Haiti earthquake in 2010. So, I was working on establishing standards that would ensure certain system elements were in place to support safe surgical care, even if that care is not high tech or expensive. But my main focus during that period was human resource capacity building. That work culminated in me going to Rwanda, where I trained surgeons under the Human Resources for Health Program at the University of Rwanda. One of the objectives of the programme was to train doctors in their own context with the resources that were available to them and also to encourage the doctors we trained to stay in Rwanda.

GH: Because they were leaving once they became qualified?

KC: That’s right. One of the biggest challenges to developing capacity in Rwanda was recruiting, training and retaining surgeons. Of course, this is a problem faced by many countries, especially countries that don’t have their own postgraduate surgical training programmes. Once doctors go abroad to train, they often don’t come home.

GH: How can countries address this issue?

KC: One way is to upskill the surgical workforce in rural hospitals, people who are not fully-fledged surgeons, and to focus on the development of a specific package of procedures that can be executed by these cadres. Taking that approach can overcome the obstacles to both training and retaining, while increasing access to essential surgical services.

GH: Has anyone drawn up the kind of package that would be suitable?

KC: It’s still under discussion. The World Bank came up with 44 essential surgical procedures as part of their *Disease control priorities*, 28 of which were to be done at district hospital level. I’m not entirely convinced that the list can work for all countries. For example, emergency abdominal operations, such as intestinal repairs and hysterectomies may not work for district hospitals. Some of the procedures that have been discussed are reduction of closed fractures, lower limb amputations, caesarean section, hernia surgery and incision and drainage of abscesses. The key is to focus on the simplest procedures with the biggest impact, but in the end, I think that countries should come up with their own national surgical plans based on what they are capable of delivering at the district level and what kind of training program they are willing to roll out.

“The key is to focus on the simplest procedures with the biggest impact.”

GH: What about concerns regarding quality of service delivery?

KC: Surgical delivery at district hospitals has to be coupled with adequate training, monitoring of outcomes and co-management by surgeons from referral hospitals. In terms of improved access, if persons with surgical conditions can access services closer to home, they may be more likely to seek care. Also, it is less of a financial and social burden to be near to family and social support. There are big benefits for the health system too.

GH: Can you say more about that?

KC: Treatment bottlenecks are a big issue at regional and tertiary hospitals in South Africa. It’s not uncommon for people with acute appendicitis to wait two days for their operation, in which time perforation often occurs. Here at the university we recently did an audit of a Cape Town health district which showed that one third of abscess drainage and appendectomies were performed at tertiary hospitals. If these procedures could be performed at lower level hospitals, then tertiary hospitals could do more complex procedures, such as coronary bypass operations.

GH: Much of the recent discussions regarding universal coverage has focused on primary health care delivery. Is the importance of surgery in that level of care being reflected?

KC: I don’t think so, and I think the main reason for that is the misperception that surgery requires too much training and too many resources. But I think if we can establish a basic package of surgical care that can be delivered at the district hospital level we can change the conversation.

GH: Is that what you are doing at Stellenbosch University?

KC: The Centre for Global Surgery at Stellenbosch University is informing the conversation and providing evidence-based solutions to help health managers make good decisions around increasing safe and equitable surgical access. I hope we are also focusing the conversation in ways that lead to action. We need research that follows through to implementation. We also need to engage community and patient groups to ensure that surgical care is their priority as well.

GH: What do you hope to achieve between now and the deadline for the Sustainable Development Goals (SDGs)?

KC: I hope that we can convince national and international policymakers of the importance of surgical care in achieving the SDGs. The *Lancet* Commission’s reporting that 28–32% of the global burden of disease is amenable to surgical care, obviously helps to raise this point, but we also need to recognize that essential surgery is deliverable at the primary health-care level.

